# Performance Improvement of a Nonvolatile UV TD Sensor Using SAHAOS with a High Temperature Annealed, Partially Nano-Crystallized Trapping Layer

**DOI:** 10.3390/s19071570

**Published:** 2019-04-01

**Authors:** Wen-Ching Hsieh

**Affiliations:** Opto-Electronic System Engineering Department, Minghsin University of Science and Technology, Xinfeng 30401, Taiwan; wchsieh@must.edu.tw; Tel.: +886-936-341-710

**Keywords:** nano, SOHOS, UV, TD, sensor

## Abstract

This study shows that a silicon–aluminum oxide–hafnium aluminum oxide-silicon oxide–silicon capacitor device with a high temperature pre-metal-anneal-treated and partially-nanocrystallized hafnium aluminum oxide, (hereafter PNC-SAHAOS) can successfully increase the performance of a nonvolatile ultraviolet radiation total dose (hereafter UV TD) sensor. The experimental results show that the UV-induced threshold voltage V_T_ shift of PNC-SAHAOS was 10 V after UV TD 100 mW·s/cm^2^ irradiation. The UV-induced charge density of PNC-SAHAOS is almost eight times that of amorphous silicon–aluminum oxide–silicon nitride–silicon dioxide–silicon SANOS. Moreover, the charge fading rate of ten-years retention on PNC-SAHAOS, even at 85 °C, is below 10%. At 85 °C, the charge fading rate of ten-years retention on amorphous SANOS is almost twice that on PNC-SAHAOS. These results strongly suggest that PNC-SAHAOS could be the most promising candidate for next-generation nonvolatile UV TD sensor technology.

## 1. Introduction

Ultraviolet (UV) radiation is used in many fields and plays an important role in fields such as optical sensing, industrial and commercial testing, biomedical technology, medical diagnosis and treatment, industrial manufacturing, semiconductor manufacturing, defense technology, and environmental protection. In the future, the UV TD measurement market will be huge, but there is no good way to make it popular.

Conventional semiconductor radiation intensity sensing elements are semiconductor photodiodes (PIN), [1] and conventional radiation total dose (TD) sensing elements are thermal luminescent dosimeters (TLD) [2], but they still have some drawbacks that need to be improved. The PIN device has the advantages of a small size, light weight, and high sensitivity. However, the PIN component is a radiation intensity sensing component, not a radiation TD sensing component. The TLD component is a non-volatile radiation TD sensing component, but the TLD’s TD record is not easily readable, and reading of the TLD’s TD record requires a sophisticated thermal illumination detection system. Recently, metal-oxide-semiconductor- (MOS) type radiation dose sensors have been studied. However, the radiation dose record of these MOS-type radiation dose sensors cannot be saved for a long period, even at room temperature [3,4]. The silicon–silicon oxide–silicon nitride–silicon oxide–silicon- (SONOS) type nonvolatile memory is chosen as one of the most promising candidates for next generation nonvolatile memory due to its coupling-free and MOS-like structure. However, a SONOS-like capacitor device has also been shown to be suitable for nonvolatile UV TD sensor applications [5]. UV radiation causes a charging effect of the SONOS device, and the UV-induced charging effect of the SONOS is associated with UV TD. The UV TD information can be permanently stored and accumulated in the non-volatile SONOS device. Even after 10 years of retention, the UV-induced charge of the SONOS-like device maintains good reliability at room temperature. For SONOS-type non-volatile UV TD sensors, the electrons in the capture layer find it difficult to escape to the control gate due to the relatively high barrier height of the thick SiO_2_ barrier oxide. Therefore, UV-induced negative charges are permanently accumulated and stored in the capture layer [5].

In advanced processes, high-k materials exhibit a superior performance compared to standard silicon nitride as a charge trap layer for SONOS-type nonvolatile memories due to their scalability and low voltage applications. A silicon–silicon dioxide–hafnium oxide–silicon dioxide–silicon- (SOHOS) like capacitor device has also been shown to be suitable for nonvolatile UV TD sensor applications in previous studies [6]. However, the improvement of the UV-inductive charging response performance and charge retention reliability for SOHOS-like capacitor UV TD sensors have not been well-studied. In this study, a high temperature pre-metal-annealing (PMA) treated silicon–aluminum oxide–hafnium aluminum oxide-silicon oxide–silicon capacitor device with a partially nanocrystallized trapping layer (hereafter PNC-SAHAOS) is proposed as an improved nonvolatile UV TD sensor. The performance of some devices, such as non-volatile UV TD sensors, including silicon–aluminum oxide–silicon nitride–silicon dioxide–silicon (SANOS) and silicon–aluminum oxide–hafnium aluminum oxide-silicon oxide–silicon capacitor devices (hereafter SAHAOS) with different crystal structure trapping layers, is compared. The results show that the PNC-SAHAOS device can significantly improve the UV-inductive charging response and charge retention characteristic of the UV TD sensor in this study. Table 1 is a comparison of the UV sensors described in this paragraph.

**Figure 1:**
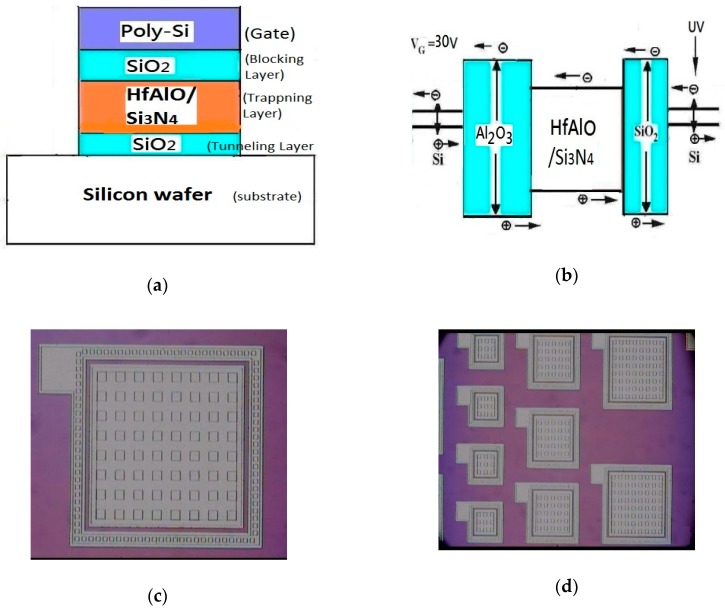
(**a**) Cross-sectional view of SAHAOS/SANOS capacitor device; (**b**) UV-induced charge generation and capture in SAHAOS/SANOS capacitor. (**c**) OM image of SAHAOS/SANOS capacitor with an area of 100 × 100 μm^2^. (**d**) OM image of SAHAOS/SANOS capacitors with an area of 100 × 100 μm^2^, 200 × 200 μm^2^, and 300 × 300 μm^2^.

The author of this paper has proposed a mechanism for UV-induced charging in a SAHAOS/SANOS UV TD sensor. The application of both UV and positive gate voltage (hereafter PGV) to the SAHAOS/SANOS capacitor device at the same time is necessary for the writing of UV TD information. UV radiation induced the ionized electron-hole pairs in the silicon substrate of the SAHAOS/SANOS capacitor device. The survival yield of UV-induced electron-hole pairs depends on the PGV of the SAHAOS/SANOS device, due to the easy recombination of UV-induced electron-hole pairs [7]. These UV-induced charges are swept over the Si-SiO_2_ potential barrier by an electric field of the SAHAOS/SANOS device, and injected into the hafnium aluminum oxide (HfAlO) trapping layer, and some of these charges are captured by the HfAlO/Si_3_N_4_ trapping layer. The accumulation of UV-induced charges changes the V_T_ of the SAHAOS/SANOS device, and the UV-induced threshold voltage V_T_ shift depends on the UV TD absorbed by the SAHAOS/SANOS device. However, the trapped UV-induced charge finds it difficult to escape to the control gate owing to the thick and relatively large barrier height of the SiO_2_ blocking oxide. Finally, the trapped UV-induced charges are permanently accumulated in the trapping layers of the SAHAOS/SANOS capacitor device. In order to erase the UV TD data information in the SAHAOS/SANOS UV TD sensor, a negative gate voltage (hereafter NGV) is applied on SAHAOS/SANOS to erase the UV inductive-negative-charge in the HfAlO/Si_3_N_4_ capture layer and restore the SAHAOS/SANOS UV-TD-sensor to its original pre-irradiated state. This is the UV–induced charging, discharging, and charge retention process in the nonvolatile SAHAOS/SANOS UV TD sensor. In Figure 1a, the cross-sectional view of the nonvolatile SAHAOS/SANOS UV TD sensor is shown. Moreover, the UV-induced charge generation and capture states of the nonvolatile SAHAOS/SANOS UV TD sensor are shown in Figure 1b.

## 2. Experimental Section

### 2.1. Sensor Fabrication

To compare the nonvolatile UV TD sensor performance, silicon–aluminum oxide–hafnium aluminum oxide-silicon oxide–silicon (hereafter SAHAOS) with different temperature PMA treatment and standard silicon–aluminum oxide–silicon nitride–silicon dioxide–silicon (SANOS) capacitor devices were prepard for this study. The SAHAOS/SANOS (SONOS-like) devices are five-layer structures in this study: (1) Substrate: SAHAOS/SANOS capacitor structures were fabricated on p-type Si <100> substrate with a resistivity of 15–25 ohm-cm; (2) charge tunneling layer: the silicon oxide SiO_2_ (3~7 nm) was used for the tunneling oxide; (3) charge storage layer: hafnium aluminum oxide (HfAlO) and silicon nitride (Si_3_N_4_) films (50~150 nm) were deposited as the charge-trapping oxide; (4) charge blocking layer: aluminum oxide Al_2_O_3_ (5~15 nm) was deposited as the blocking oxide; and (5) gate electrode: poly silicon Si (100~400 nm) was used for the gate material.

### 2.2. Sensor Fabrication

There are five fabrication steps for the five-layers-structure SONOS-like devices used in this study: (1) Tunneling oxide: the tunneling oxide SiO_2_ was thermally grown at 925 °C by an advanced clustered vertical furnace (ASM A-400) as tunneling oxide for SAHAOS and SANOS devices; (2) trapping oxide: after the tunneling oxide formation, hafnium aluminum oxide HfAlO films were deposited by a metal organic chemical vapor deposition MOCVD system (AIXTRON Tricent 800016) at 400~550 °C as the charge-trapping layers. There were two precursors used for HfAlO films deposition: Hf[OC(CH_3_)_3_]_4_ (Sigma-Aldrich, Inc.) and aluminum isopropoxide Al(OC_3_H_7_)_3_ (Sigma-Aldrich, Inc.). By controlling the mixing ratio of the two precursors during the deposition process, the Al composition ratio in HfAlO is about 10%~40%. For standard SANOS devices, after the tunneling oxide was formed, silicon nitride Si_3_N_4_ (hereafter, nitride) was deposited as the charge-trapping layer by low-pressure chemical vapor deposition LPCVD (SVCS Furnace system); (3) blocking oxide: for the top oxide of SAHAOS/SANOS_,_ the aluminum isopropoxide precursor Al(OC_3_H_7_)_3_ (Sigma-Aldrich, Inc.) was used for Al_2_O_3_ film deposition by MOCVD; (4) PMA: the pre-metal annealing (PMA) at different temperatures was treated with a rapid thermal annealing (RTA) process in N_2_ ambient (KORONA RTP 800) after the gate dielectric deposition for the SAHAOS/SANOS device; (5) gate electrode: the poly silicon (200–400 nm) was formed by low-pressure chemical vapor deposition LPCVD (ASM Vertical Furnace system) as the control gate material after the PMA process for the SAHAOS/SANOS device. In Table 2, the various SAHAOS devices prepared with various PMA temperature conditions are listed. To compare the UV TD nonvolatile sensor performance for SAHAOS devices with different PMA temperatures and SANOS, three types of SAHAOS with PMA at different temperatures and one SANOS were prepared: (1) standard SANOS with 900 °C 30 s PMA (STD-SANOS); (2) SAHAOS with 900 °C 30 s PMA (hereafter SAHAOS-T1); (3) SAHAOS with 1000 °C 30 s PMA (hereafter SAHAOS-T2); and (4) SAHAOS with 1100 °C 5 s PMA (hereafter SAHAOS-T3). The poly silicons (100–400 nm) were formed by low-pressure chemical vapor deposition (LPCVD) as the control gate. Aluminum Al was sputtered by a Duratek sputter machine on the bottom of the Si substrate to form an ohmic contact. In this study, three types of SAHAOS device and one SANOS capacitor device have the same thickness of the tunneling oxide, trapping oxide, and blocking oxide layer for the nonvolatile UV TD sensor performance comparison.

### 2.3. Sensor UV TD Information Measurement

There are three steps for the SONOS-like nonvolatile sensor UV TD information measurement in this study: (1) Reset record: prior to writing of the UV TD information, a negative gate voltage (NGV) V_G_ = −40 V is forced on the SAHAOS and SANOS capacitor device to release the trapped native negative charge from the HfAlO and Si_3_N_4_, and the SAHAOS and SANOS capacitor devices are erased to a null state; (2) writing record: during the writing of UV TD information, both UV and positive gate bias (PGV) are applied to the SAHAOS and SANOS capacitor devices at the same time. The CNI MLL-III-405 UV LED with a 405 nm wavelength is used for the UV radiation source. Table 3 presents a list of various UV radiation and various PGV conditions applied simultaneously on the SAHAOS and SANOS capacitor devices; (3) reading record: the V_T_ variation can be calculated from the C_G_-V_G_ curves shifting after UV TD irradiation. The gate capacitance vs. various gate voltages (C_G_-V_G_) curves were measured by a HP4284 parameter analyzer at room temperature before and after UV TD irradiation. The gate leakage current vs. gate voltage (I_G_-V_G_) curves for an SAHAOS and SANOS capacitor device were measured at room temperature using a computer-controlled Agilent HP4156A parameter analyzer before and after UV TD irradiation.

### 2.4. Sensor Material Analysis

**Figure 2:**
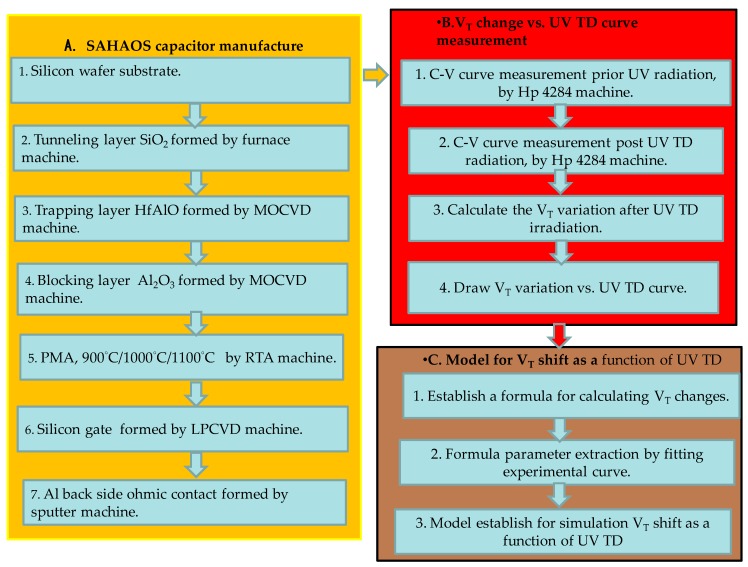
Experimental and simulation approach.

There are three steps for sensor material analysis in this study: (1) OM: Figure 1c,d show the optical microscope (OM) top view image of an SAHAOS and SANOS capacitor’s pattern; (2) TEM: in this paper, transmission electron microscopy (TEM) (JEOL JEM-2010F) was used for HfAlO crystallization analysis of the various SAHAOS devices prepared with various PMA temperature conditions; (3) XRD: X-ray diffraction analysis (XRD) (PANalytical X’Pert Pro) was used for temperature-dependent crystallization analysis of various HfAlO films prepared with various PMA temperature conditions. Figure 2 shows the flow including the experimental and simulation approach.

## 3. Results

### 3.1. UV-Induced V_T_ Shift in SAHAOS

**Figure 3:**
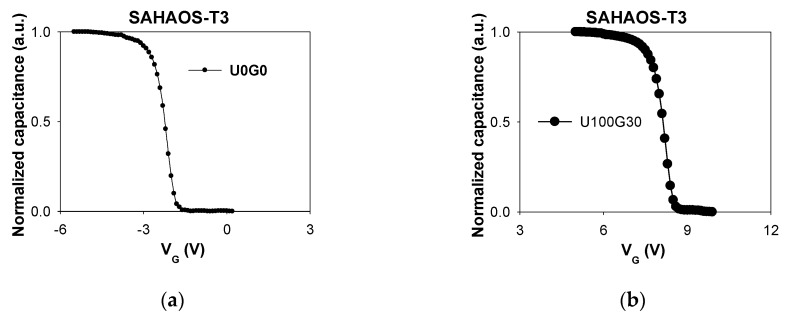
C_G_-V_G_ curve for an SAHAOS-T3 device (**a**) before UV irradiation; (**b**) after 100 mW·s/cm^2^ UV TD irradiation at PGV 30 V.

Figure 3a illustrates a C_G_-V_G_ curve for an SAHAOS-T3 capacitor before UV irradiation. Figure 3b demonstrates a C_G_-V_G_ curve for a post-UV-irradiated SAHAOS-T3 capacitor device after 100 mW·s/cm^2^ UV irradiation under PGV 30 V. The C_G_-V_G_ curve of the SAHAOS-T3 capacitor shifted to the right after UV TD up to 100 mW·s/cm^2^ at PGV 30 V, as illustrated in Figure 3b.

**Figure 4:**
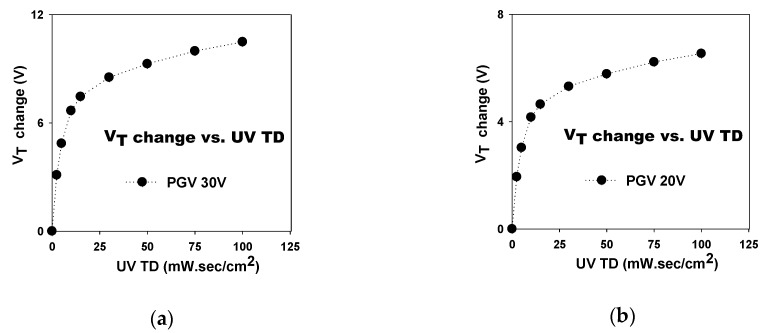
The dependence of the UV-induced V_T_ increase on UV TD for an SAHAOS-T3 capacitor (**a**) at PGV 30 V; (**b**) at PGV 20 V.

The V_T_ increase as a function of UV TD for the SAHAOS-T3 device at 30 V PGV is illustrated in Figure 4a. Consistent with previous studies, the increase in V_T_ in SAHAOS-T3 was associated with an increase in UV TD, but when UV TD was greater than 30 mW·s/cm^2^, V_T_ increased more slowly [6]. For the SAHAOS-T3 device, the dependence of the V_T_ shiftting on UV TD at PGV 30 V is more significant than that at PGV 20 V, as shown in Figure 4a,b, respectively.

**Figure 5:**
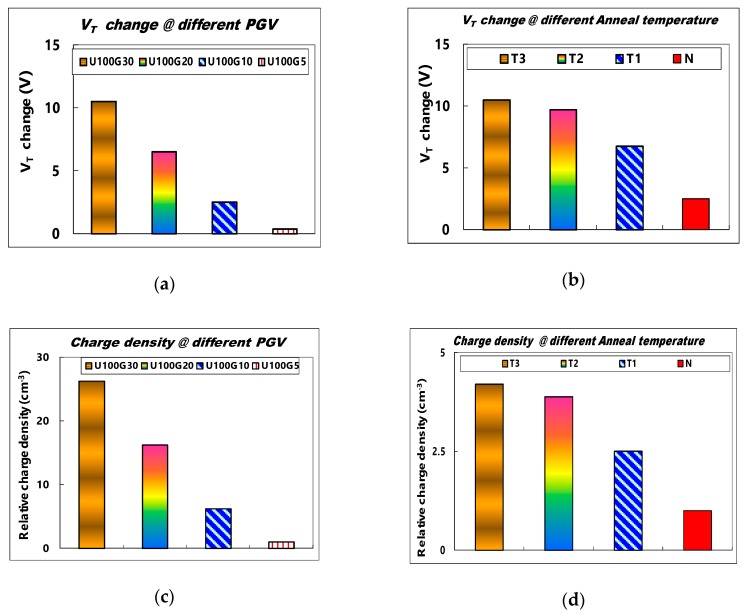
(**a**) The UV-induced V_T_ change comparison of SAHAOS-T3 capacitor devices at different PGVs after 100 mW·s/cm^2^ UV TD irradiation; (**b**) the UV-induced V_T_ change comparison for various SAHAOS and SONOS capacitor devices with different PMA temperatures after the U100G30 irradiation condition; (**c**) the relative UV-induced charge density comparison of SAHAOS-T3 capacitor devices at different PGVs after 100 mW·s/cm^2^ UV TD irradiation; (**d**) the relative UV-induced charge density comparison for various SAHAOS and SONOS capacitor devices with different PMA temperatures after the U100G30 irradiation condition.

Figure 5a shows a comparison of V_T_ changes in the SAHAOS-T3 device after the fixed 100 mW·s/cm^2^ UV TD irradiation at different PGVs. In Table 3, a list of symbols for various UV and PGV conditions on the SAHAOS-T3 device is listed. Under UV TD 100 mW·s/cm^2^ irradiation, the V_T_ change of SAHAOS-T3 is very significant at PGV 30 V compared to those at different PGVs under UV TD 100 mW·s/cm^2^ irradiation, as shown in Figure 5a. It is noted that even with 100 mW·s/cm^2^ UV TD irradiation, the V_T_ change of SAHAOS-T3 at 5 V PGV is very small compared to the those at different PGVs, as shown in Figure 5a.

Figure 5c shows the relative charge density comparison of SAHAOS-T3 devices after 100 mW·s/cm^2^ UV TD irradiation at different PGVs. It is worth noting that, as shown in Figure 5c, the UV-induced charge density of SAHAOS-T3 at PGV 30 V is almost 25 times greater than that at PGV 5 V under UV TD 100 mW·s/cm^2^ illumination. As shown in Figure 5a, the change in V_T_ of the SAHAOS-T3 device is negligible only under UV illumination in the absence of PGV. By applying different PGVs during the UV illumination, the sensitivity of the SAHAOS devices is adjustable, which makes them suitable for various applications.

Figure 5b shows a comparison of the V_T_ change for various SAHAOS devices with different PMA temperatures after the U100G30 irradiation condition. Table 2 shows a list of symbols for SAHAOS devices prepared with various PMA temperatures. It is important to note that, under the U100G30 irradiation condition, the UV-induced V_T_ changes of SAHAOS-T3 and SAHAOS-T2 are very significant compared to those of SAHAOS-T1 and STD-SAONOS, as shown in Figure 5b. Figure 5d shows the relative UV-induced charge density for various SAHAOS and STD-SANOS devices with different PMA temperatures after the U100G30 irradiation condition. Under the U100G30 irradiation condition, it is noted that the UV-induced V_T_ change of SAHAOS-T3 with 1100 °C 5 s PMA is almost four times greater than that of STD-SANOS with 900 °C 30 s PMA. Meanwhile, the UV-induced V_T_ change of the SAHAOS-T3 is almost two times that of the SAHAOS-T1 with 900 °C 30 s PMA under the U100G30 irradiation condition. Moreover, the UV-induced V_T_ change of SAHAOS-T3 with 1100 °C 5 s PMA is almost 1.1 times SAHAOS-T2 with the 1000 °C 30 s PMA under U100G30 irradiation, as shown in Figure 5b. It is also noted that the UV-induced charge density of SAHAOS-T3 with 1100 °C 5 s PMA is almost eight times greater than that of STD-SANOS with 900 °C 30 s PMA under the U100G30 irradiation condition. The SAHAOS-T3 and SAHAOS-T2 both demonstrate higher degrees of UV-induced V_T_ shift and UV-induced charge density than the SAHAOS-T1 and STD-SANOS devices.

**Figure 6:**
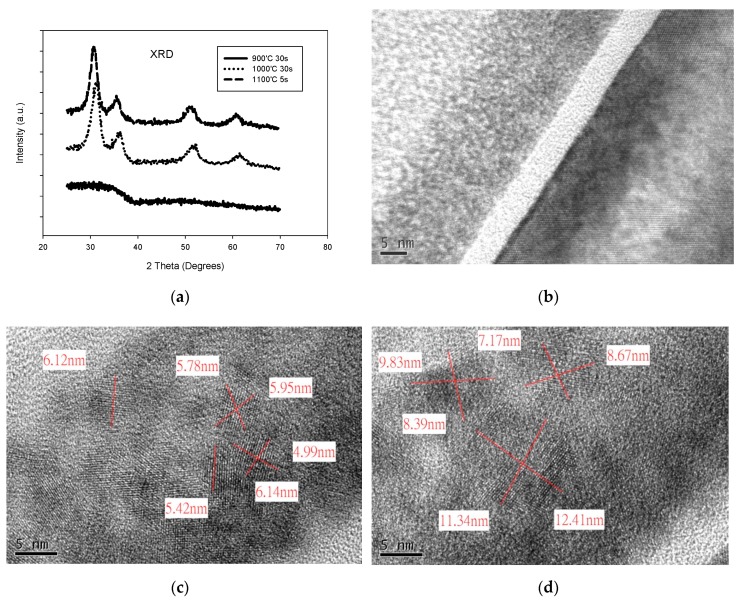
(**a**) XRD analysis comparison of HfAlO films with different anneal temperatures; (**b**) TEM image of SAHAOS capacitor with 900 °C 30 s PMA; (**c**) TEM image of SAHAOS capacitor with 1000 °C 30 s PMA; (**d**) TEM image of SAHAOS capacitor with 1100 °C 5 s PMA.

An X-ray diffraction (XRD) analysis comparison of HfAlO films with different PMA temperatures is shown in Figure 6a. The XRD analysis shows the temperature-dependent crystallization of HfAlO, as presented in Figure 6a. A transmission electron microscopy (TEM) comparison of HfAlO films with different PMA temperatures is shown in Figure 6b–d. It can be seen from the XRD and TEM results that the HfAlO film is almost still amorphous, even after annealing at 900 °C 30 s, and is partially nano crystallized after annealing at both 1000 °C 30 s and 1100 °C 5 s. Further, it seems that the HfAlO film annealed at 1100 °C for 5 s has a larger number of partial nanocrystals than the HfAlO film annealed at 1000 °C for 30 s, as shown in Figure 6. The Al composition ratios in HfAlO of SAHAOS-T3, SAHAOS-T2, and SAHAOS-T1 devices are all the same, with a value of about 30% in this study. The trap density of the HfO_2_ trap layer of the SOHOS device can be increased by doping a suitable Al content into the HfO_2_ charge trap layer [8,9,10,11,12,13].

### 3.2. Gate Leakage Current Comparison

**Figure 7:**
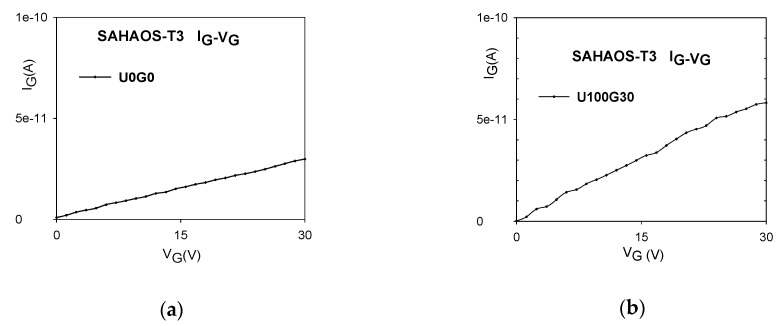
The gate leakage current at PGV 30 V for an SAHAOS-T3 capacitor device (**a**) before UV irradiation; (**b**) after U100G30 irradiation.

The I_G_-V_G_ curves for a SAHAOS-T3 capacitor device before and after the U100G30 irradiation condition are shown in Figure 7a,b, respectively. As demonstrated in Figure 7a,b, the gate oxide leakage current at PGV 30 V for the SAHAOS-T3 capacitor device did not increase significantly after the U100G30 irradiation condition.

**Figure 8:**
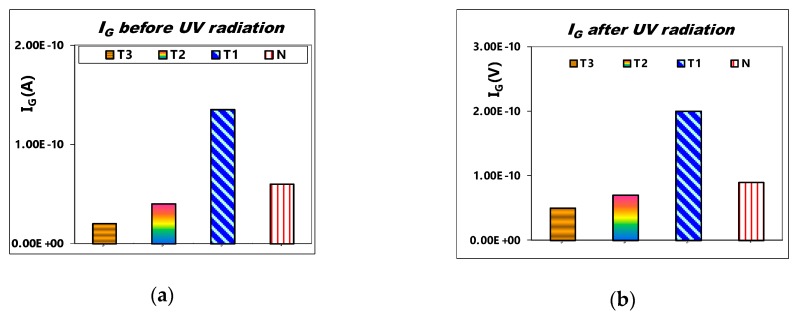
(**a**) The gate-current comparison at V_G_ 30 V for various SAHAOS with different PMA temperatures before the UV irradiation condition; (**b**) The gate-current at V_G_ 30 V for various SAHAOS with different PMA temperatures after the U100G30 irradiation condition.

Figure 8a shows the comparison of I_G_ at V_G_ = 30 V for various SAHAOS with different PMA temperatures before UV irradiation. Figure 8b demonstrates the comparison of I_G_ at V_G_ = 30 V for various SAHAOS with different PMA temperatures after UV TD 100 mW·s/cm^2^ irradiation. As illustrated in Figure 8a,b, the gate dielectric leakage current of the SAHAOS-T3 and SAHAOS-T2 devices at V_G_ = 30 V before and after UV irradiation is significantly improved compared to the SAHAOS-T1 and STD-SANOS devices.

### 3.3. V_T_ Stability vs. Retention Time

**Figure 9:**
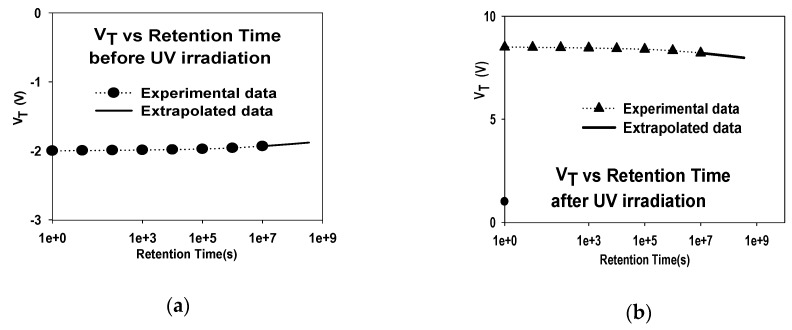
The V_T_ room temperature retention characterization curve for an SAHAOS-T3 device: (**a**) before UV irradiation; and (**b**) after U100G30 irradiation.

The V_T_ room temperature retention characterization for an SAHAOS-T3 device before and after the U100G30 irradiation condition is demonstrated in Figure 9a,b, respectively. As shown in Figure 9a, before UV irradiation, the native negative charge naturally tunnels to the HfAlO capture layer, and the V_T_ of the SAHAOS-T3 device increases with time. As shown in Figure 9b, after UV irradiation, the UV-induced negative charge escapes from the HfAlO capture layer, and the V_T_ of the SAHAOS-T3 device decreases over time [5,6]. In addition, the V_T_ retention loss of the SAHAOS-T3 device is less than 5% after 10 years of retention.

**Figure 10:**
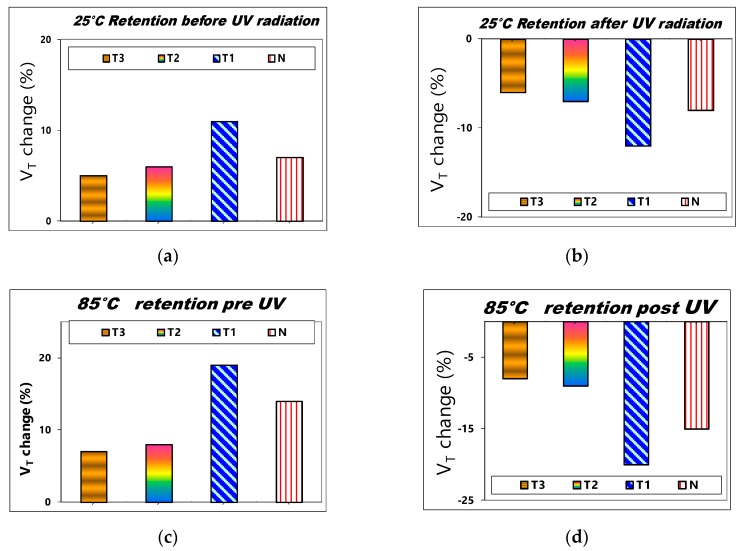
The comparison of V_T_ change after a 10-years retention for various SAHAOS and SANOS with different PMA temperatures (**a**) before UV irradiation at 25 °C; (**b**) after UV100G30 irradiation at 25 °C; (**c**) before UV irradiation at 85 °C; (**d**) after UV100G30 irradiation at 85 °C.

Figure 10a,b illustrate the comparison of the 25 °C charge retention characteristics of 10 years for various SAHAOS devices with different PMA temperatures before UV irradiation and after U100G30 irradiation. As illustrated in Figure 10a,b, the 25 °C charge 10-year retention performances of the PNC-SAHAOS (SAHAOS-T3, SAHAOS-T2) devices are significantly better than that of the SAHAOS-T1 device.

The 85 °C 10-year charge retention reliability properties of comparison for various SAHAOS and SANOS devices are shown in Figure 10c,d. The PNC-SAHAOS device had good charge retention at 25 °C and 85 °C, and no significant charge loss/gain was observed. However, the SANOS device only exhibits good charge retention at 25 °C, but exhibits poor charge retention and significant charge loss/gain at 85 °C. The PNC-SAHAOS device had good charge retention at different temperatures in comparison with the SANOS device, which could not act properly at higher temperatures.

### 3.4. Model for UV-Induced V_T_ Shift

In order to simulate the V_T_ shift of SAHAOS devices after UV radiation, the author proposed a model (hereafter called HWC-UV model) derived from the prior studies of the HWC model [14]. The HWC-UV model is used to simulate the V_T_ shift of a SAHAOS-T3 device after various UV TD irradiation at different PGVs, and the formula for the model is as follows:

deltaV_T_(D) = V_T_(D) − V_T_(0) = [(V_G_ − V_o_)/A]log(t*D) (1)

In this equation, D represents the UV TD (mW·s/cm^2^), V_T_(D) is the V_T_ after UV TD irradiation, V_T_(0) is the V_T_ before UV radiation, and V_G_ is the PGV. The author used experimental curve fitting to derive the three parameters V_o_, t, and A of the HWC-UV model. t is the sum of emission and capture constant of electrons and holes. V_o_ and A are the constants for specific devices. V_o_ is the minimum PGV for UV-induced V_T_ shifting. When V_G_ is smaller than V_o_, the insufficient electric field can not separate the UV-induced electron-hole pairs and inject the UV-induced charges over the Si-SiO_2_ potential barrier into the AHAOS gate dielectric layer from the silicon substrate. A is the charge capture constant for specific devices. V_o_ is equal to 4 Volt, t is equal to 3 cm^2^/mW·s, and A is equal to 6 for SAHAOS-T3 devices in this study. They are the optimum fitting parameters to predict the UV-induced delta V_T_(D) of the SAHAOS-T3 device. Using these optimum fitting parameters, the simulated V_T_ shift after various UV TD radiation at different PGVs can be predicted using Equation (1).

**Figure 11:**
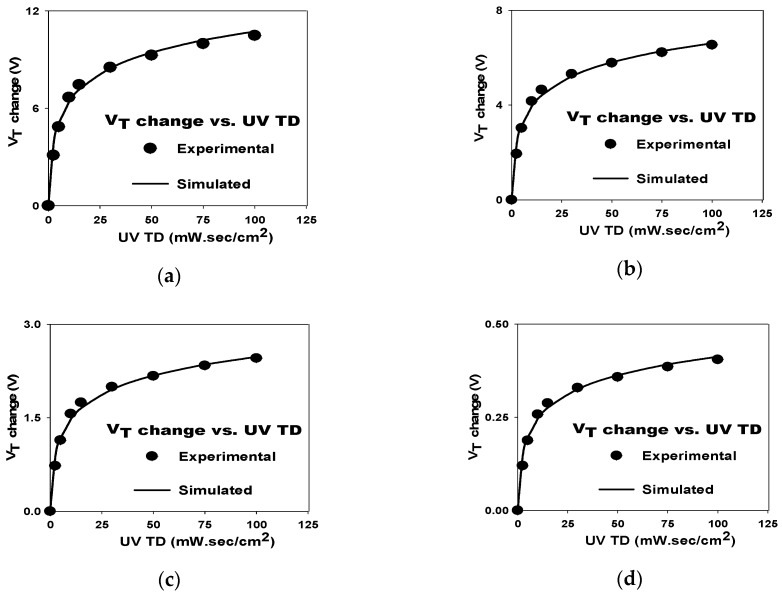
The model of V_T_ change as a function of UV TD for an SAHAOS-T3 capacitor device (**a**) under PGV 30 V; (**b**) under PGV 20 V; (**c**) under PGV 10 V; (**d**) under PGV 5 V.

The comparisons of the measured and simulated curves of the V_T_ shift verus UV TD for SAHAOS-T3 devices under four fixed PGVs are shown in Figure 11a–d. Please note that the HWC-UV model can simulate all experimental curves very well, as shown in Figure 11.

**Figure 12:**
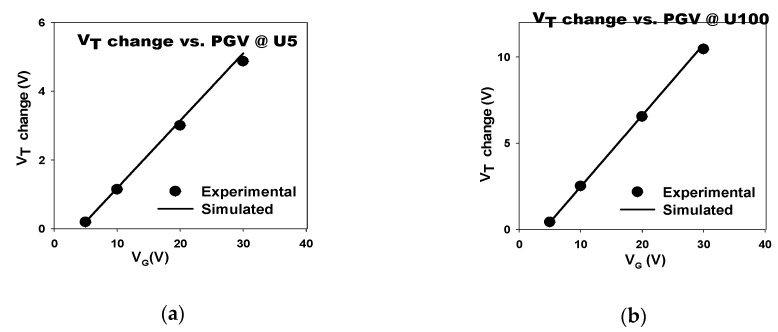
The model of V_T_ as a function of PGV under fixed UV TD for an SAHAOS-T3 capacitor device (**a**) under UV TD 5 mW·s/cm^2^ irradiation; (**b**) under UV TD 100 mW·s/cm^2^ irradiation.

Figure 12 is a comparison of the measured and simulated curves of the V_T_ shift verus PGV at fixed UV TD for a SAHAOS-T3 capacitor device, and the delta V_T_ is almost a linear function of PGV during fixed UV TD irradiation for an SAHAOS-T3 capacitor device. Figure 12 shows that the HWC-UV model can predict the curves of V_T_ shift verus PGV under fixed UV TD well.

## 4. Discussion

### 4.1. UV-Induced V_T_ Shift in SAHAOS

As illustrated in Figure 3a,b, the positive V_T_ shiftting is due to the increase in the net total UV-induced negative trapped charge accumulated in the AHAO gate dielectric layer after UV illumination at PGV. This positive V_T_ shift result is consistent with previous studies [5,6]. It is worth noting that the 100 mW·s/cm^2^ TD UV leads to a significant increase in the V_T_ for the SAHAOS-T3 at 30 V PGV by about 10.5 volts. Higher UV TD contributes to the accumulation of carriers in the inversion layer. Under UV irradiation, the applied PGV drops at the AHAO dielectric, and the density of injected electrons from the inversion layer is sufficient to cause a huge number of electrons in the AHAO trapping layer, causing the V_T_ to shift to the right.

As illustrated in Figure 5a,c, the sensitivity of SAHAOS devices is tunable by adjusting different PGVs during irradiation, which makes them suitable for various applications. For increasing PGV, there is an increase in the charge yield corresponding to a decrease in the initial recombination of UV-induced electron-hole pairs, which will tend to increase the amount of UV-induced voltage shift [7].

As illustrated in Figure 5b, the UV-induced charging responses of the SAHAOS devices with 1000 °C 30 s and 1100 °C 5 s PMA have been significantly improved, compared with the SAHAOS with 900 °C PMA and STD-SANOS devices with 900 °C PMA. Therefore, the author thinks that that the partially nanocrystallized HfAlO (hereafter PNC-HfAlO) trapping layer has a larger trap density than the amorphous HfAlO and Si_3_N_4_ trapping layer. A discrete nanocrystal structure was formed by the partial crystallization of HfAlO using the PMA process at 1000 °C 30 s and 1100 °C 5 s. However, in the partially nanocrystallized HfAlO trapping layer, HfAlO nanocrystals are embedded in amorphous HfAlO. UV-induced charges can be trapped not only in HfAlO nanocrystals, but also in amorphous regions of the HfAlO trapping layer. Therefore, it is understood that the partially nanocrystallized HfAlO trapping layer has a higher trap density than the complete amorphous HfAlO trapping layer [15,16,17,18,19,20]. The author thinks that that the nanocrystallization-induced trap generation enhances the trap density. Therefore, the SAHAOS-T3 and SAHAOS-T2 (hereafter PNC-SAHAOS) with a PNC-HfAlO trapping layer show a larger trap density compared to SAHAOS-T1 with an amorphous HfAlO trapping layer [15,16,17,18,19,20].

### 4.2. Gate Leakage Current Comparison

As illustrated in Figure 8a,b, the gate dielectric leakage current of the SAHAOS-T3 and SAHAOS-T2 devices at V_G_ = 30 V before and after UV irradiation has been significantly improved, compared with the SAHAOS-T1 and STD-SANOS device. Therefore, the author believes that the HfAlO nanocrystals highly capture localized charges and effectively suppress lateral charge migration, which can act as current leakage paths [15,16,17,18,19,20].

### 4.3. V_T_ Stability vs. Retention Time

As illustrated in Figure 10a,b, the 25 °C charge 10-years retention performances of the PNC-SAHAOS (SAHAOS-T3, SAHAOS-T2) devices are significantly better than that of the SAHAOS-T1 device. This is because the HfAlO charge-storage layer of SAHAOS-T1 is still relatively amorphous, while the HfAlO charge-storage layers of PNC-SAHAOS have already been partially nanocrystallized. The partially nanocrystallized HfAlO thin films of PNC-SAHAOS will capture highly localized charges and effectively inhibit the charge leakage path. Hence, for PNC-SAHAOS, there will be a decrease in the charge leakage path of HfAlO films, which can result in better charge retention [15,16,17,18,19,20].

The PNC-SAHAOS device had good charge retention at different temperatures in comparison with the SANOS device that cannot act properly at higher temperatures. The charge retention performance of the PNC-HfAlO charge storage layer is significantly improved compared to that with amorphous Si_3_N_4_ and amorphous HfAlO in this paper, which is likely attributed to the partially crystallized HfAlO suppressed lateral charge migration. The partially nanocrystallized HfAlO thin films of PNC-SAHAOS will capture highly localized charges and effectively inhibit the charge leakage path. Hence, for PNC-SAHAOS, there will be a decrease in the charge leakage path of HfAlO films, which can result in better charge retention [15,16,17,18,19,20]. In the partially crystallized HfAlO trapping layers, nanocrystals are embedded in amorphous HfAlO; thus, injected electrons can be trapped in HfAlO nanocrystals and the amorphous HfAlO matrix. The structure with nanocrystals embedded in an amorphous dielectric matrix could suppress lateral charge migration. The partially nanocrystallized HfAlO thin films of PNC-SAHAOS will capture highly localized charges and effectively inhibit the charge leakage path. Hence, for PNC-SAHAOS, there will be a decrease in the charge leakage path of HfAlO films, which can result in better charge retention. Moreover, the improvement of charge-retention performance is primarily due to the annihilation of shallower charge traps and increase of deeper charge traps in the HfAlO trapping layer. The PNC-SAHAOS devices with deeper charge traps in the PNC-HfAlO trapping layer showed better charge-retention reliability characteristics than the SAHAOS-T1 and SANOS devices with shallower charge traps in the amorphous HfAlO and Si_3_N_4_ trapping layer.

## 5. Conclusions

Compared with traditional UV intensity sensing elements, the SOHOS-type non-volatile UV TD sensor has the following advantages: (1) Small size and light weight; (2) good correlation between accumulated UV-inductive charge and UV TD; (3) UV TD recording can be permanently accumulated in the device; (4) UV TD recording can be erased; (5) devices with high-k technology can be integrated into the next generation IC chip; (6) data reading equipment is simple and convenient; and (7) the sensitivity of a UV TD sensor is adjustable [6].

The UV-induced charging response and the charge retention characteristics of the PNC-SAHAOS capacitor devices with 1000 °C and 1100 °C PMA have been significantly improved, compared with the amorphous SAHAOS-T1 and SANOS devices with 900 °C PMA. The PNC-HfAlO charge storage layer containing nanocrystals formed in an amorphous HfAlO matrix shows both crystalline and amorphous HfAlO properties. UV-induced charges can be trapped not only in nanocrystals, but also in amorphous matrices. Therefore, the author thinks that the PNC-HfAlO trapping layer has a larger trap density than the amorphous HfAlO and Si_3_N_4_ trapping layer. Furthermore, the author considers that the PNC-HfAlO trapping layer has suppressed lateral charge migration compared to the amorphous Si_3_N_4_ and HfAlO trapping layer. It is true that a higher PMA temperature (>900 °C) is necessary to form the high-κ Hf-based PNC-HfAlO to enhance the nonvaltile UV TD sensor performance in this paper. These results strongly suggest that PNC-HfAlO is a promising charge-trapping structure for next-generation nonvolatile SONOS-type UV TD sensor technology.

## Figures and Tables

**Table 1:** Comparison of UV sensors.

Sensor Device Abbreviation	Device Materials Composition	Characterization	Reference
PIN	SiC	Cannot measure UV TD, only can measure UV intensity	[1] Sze, S.M. 2006.
TLD	Er_2_O_3_ doped ZrO_2_	UV TD record is not easily readable	[2] Hsien, W.C. 1994.
MOS	Si-SiO_2_-Si	Stability with time after irradiation is very bad	[3] Pejović, M.M. 2016. [4] Ho, W.S. 2008.
SONOS	O doped Si-SiO_2_-Si_3_N_4_-SiO_2_-Si	Sensitivity to irradiation and stability with time after irradiation can been improved	[5] Jong, F.C. 2018.
F-SOHOS	F doped Si-SiO_2_-HfO_2_-SiO_2_-Si	Sensitivity to irradiation and stability with time after irradiation can been improved	[6] Hsien, W.C. 2018.
PNC-SAHAOS	1000 °C/1100 °C PMA Si-Al_2_O_3_-HfAlO-SiO_2_-Si	Sensitivity to irradiation and stability with time after irradiation are very good	This paper.

**Table 2:** SAHAOS devices prepared with various PMA processes.

Split	T1	T2	T3	N
PMA temperature (°C)	900	1000	1100	900
PMA time (s)	30	30	5	30
Trapping Layer	HfAlO	HfAlO	HfAlO	Si_3_N_4_

**Table 3:** The list of various UV TD and PGV conditions applied simultaneously on SAHAOS.

Symbol	UV TD (mW·s/cm^2^)	PGV (V)
U0G0	0 mW·s/cm^2^	0 V
U100G5	100 mW·s/cm^2^	5 V
U100G10	100 mW·s/cm^2^	10 V
U100G20	100 mW·s/cm^2^	20 V
U100G30	100 mW·s/cm^2^	30 V
